# Ameliorative effect of Mangosteen (*Garcinia mangostana* L.) peel infusion on the histopathological structures of the liver and kidney of rats (*Rattus norvegicus* Berkenhout, 1769) after H_2_O_2_ induction

**DOI:** 10.14202/vetworld.2021.1579-1587

**Published:** 2021-06-18

**Authors:** J. R. A. Rusman, S. A. Sundari, A. Nuriliani, H. T. Saragih

**Affiliations:** 1 Alumni of Faculty of Biology, Universitas Gadjah Mada, Yogyakarta 55281, Indonesia; 2 Laboratory of Animal Structure and Development, Faculty of Biology, Universitas Gadjah Mada, Yogyakarta 55281, Indonesia

**Keywords:** antioxidant, female rat, H_2_O_2_, histological structure, mangosteen peel infusion

## Abstract

**Background and Aim:**

Hydrogen peroxide (H_2_O_2_) is a free radical, widely use as a food preservative, may cause adverse effects to the body. Mangosteen contains various antioxidants may scavenge free radical produced by H_2_O_2_. This study examined the effects of mangosteen peel infusion on the liver and kidney of rats after H_2_O_2_ induction.

**Materials and Methods:**

Thirty-six female Wistar rats were divided into six groups: Negative control, positive control (1% H_2_O_2_), as well as mangosteen peel infusion groups at a concentration of 0.25; 0.5; 1, and 2%. H_2_O_2_ induction was administered orally every day for 2 months followed by mangosteen peel infusion treatment (1 month) after H_2_O_2_ induction for 1 month. At the end of the experiment, the rats were sacrificed. The liver and kidney of each rat were collected for reactive oxygen species (ROS) and histopathological examinations. Furthermore, blood was collected for creatinine examination.

**Results:**

H_2_O_2_ induction caused the highest ROS level elevation in the positive control group which was treated with H_2_O_2_ only. Meanwhile, 2% of mangosteen peel infusion returned the ROS levels to normal. It was also observed that mangosteen peel infusion restored creatinine levels to normal. Furthermore, 2% of mangosteen peel infusion improved the histopathological structure of the liver and kidneys after H_2_O_2_ induction.

**Conclusion:**

Mangosteen peel infusion especially at a concentration of 2% has the potential to improve liver and kidney structure and functions after H_2_O_2_ induction.

## Introduction

The addition of preservatives in food and beverages is expected to prolong the consumption duration. However, some preservatives cause adverse effects on the body. H_2_O_2_ is a widely used preservative, especially in pasteurizing milk, freezing food, and eliminating mycotoxins found in food [1-3]. H_2_O_2_ has a high oxidizing ability which causes oxidative stress. This oxidative stress causes adverse effects to the body such as erythema (redness) in the oral mucosal tissue, hematemesis (blood vomiting), wounds on the entire surface of the stomach, and erosion on the surface of the duodenum [4]. Abdullah *et al*. [5] stated that H_2_O_2_ decreased kidney function which was characterized by glomerular atrophy.

Mangosteen (*Garcinia mangostana* L.) is a native fruit from the Southeast Asia region which possesses antioxidant characteristics [6]. Instead of its fruit, the mangosteen peel could be processed as a beverage [7]. Mangosteen is traditionally utilized as a supplement to relieve chronic diseases connected to oxidative stress such as diabetes, neurodegenerative, and cardiovascular diseases [8]. Miguel [9] observed that antioxidants such as anthocyanins have phenolic hydroxyl groups that function as free-radical scavengers. Furthermore, peroxidation inside of the cell can be inhibited by phenolic hydroxyl groups. This group interrupts serial reaction in oxidation [10].

Many studies show that mangosteen peel may improve the histopathological structure of some organs such as the liver and kidney after exposure to various stressors; Widowati *et al*. [11] concluded that mangosteen peel extract prevented and improved the histopathological structure of the rats’ kidneys which was induced by monosodium glutamate. Flavonoid which was found in the peel also inhibits kidney cell destruction [11]. Adyab *et al*. [6] observed that the supplementation of mangosteen flesh occurred from a concentration of 200 mg/kg and improved liver and kidney histopathological structures of rats almost completely to their normal structures after a high-fat diet. Abood *et al*. [12] stated that treatment with mangosteen peel’s extract prevents liver cirrhosis and maintains the parenchymal architecture of hepatocytes. However, there are no previous studies that confirm the effect of mangosteen peel infusion on the organs after H_2_O_2_ induction.

This study evaluated the effect of mangosteen peel infusion on oxidative stress and histopathological structure of the liver and kidney of rats after H_2_O_2_ induction.

## Materials and Methods

### Ethical approval

Ethical approval was obtained from the Integrated Research and Testing Laboratory (LPPT), Universitas Gadjah Mada (No.0028/04/LPPT/V/2018).

### Study period and location

The study was conducted from May 2018 to May 2019. The treatment was done at Integrated Research and Testing Laboratory, Universitas Gadjah Mada. The samples were processed at Joint Research Laboratory and Animal Structure and Development Laboratory, Faculty of Biology, Universitas Gadjah Mada.

### Treatment feed formulation and preparation

Fresh ripe mangosteen was purchased from Superindo (market place), Jalan Kaliurang km 6.2, No. 51, Purwosari, Sinduadi, Sleman, Yogyakarta and identified by Prof. Dr. Purnomo, M.S., the Head of Plant Systematic Laboratory, Faculty of Biology, Universitas Gadjah Mada. The ripe mangosteen with blackish-purple color was chosen for infusion preparation. The mangosteen peels were shelled and washed with a small amount of water to maintain the secondary metabolites. 330 g of peel was added with 1000 mL of drinking water and immersed for 12 h until the color of water turned into dark red, with a fresh aroma. Afterward, 1.87% of flavonoid of mangosteen peel infusion was obtained, referred to as the stock solution. Furthermore, the working solution was prepared using concentrations of 0.25; 0.5; 1; and 2%. This infusion preparation method was adapted from Prasetyo [13] with modifications. The mangosteen peel infusion was made once every 2 weeks, stored in bottles wrapped with aluminum foil to prevent direct light, and stored at 4°C.

### Flavonoid level detection

The flavonoid levels of the mangosteen peel infusion were detected using a spectrophotometer (ultraviolet [UV]-visible 1800 Shimadzu) with a wavelength of 510 nm. The flavonoid level detection was divided into two steps: Standard curve preparation and total flavonoid test. The standard curve was prepared by mixing 100 mg of the sample with 0.3 mL of 5% sodium nitrate. After 5 min, 6 mL of 10% aluminum chloride was poured into the mixture and re-incubated for 5 min. 2 mL of 1 M sodium hydroxide was then added into the mixture. The standard concentration was made based on Table-1. The total flavonoid was detected by mixing 100 mg of sample with 0.3 mL of 5% sodium nitrate. After 5 min, 6 mL of 10% aluminum chloride was poured into the mixture and re-incubated for 5 min and 2 mL of 1 M sodium hydroxide was further added. The results showed that the total number of flavonoids was equal to 1.87% body weight (wet basis).

**Table-1:** Concentration of standard solution.

Concentration (ppm)	Liquor base(µL)	Aquabides (µL)	Volume (mL)
0	0	10000	10
1.563	15.63	9984.37	10
3.125	31.25	9968.75	10
6.25	62.5	9937.50	10
12.5	125	9875	10
25	250	9750	10
50	500	9500	10
100	1000	9000	10

### Reagents

The reagents used in this research were 1% H_2_O_2_, mangosteen peel infusion with concentrations of 0.25%, 0.5%, 1%, and 2% (based on standards by the Organization for Economic Cooperation and Development [14] and referring to the results of the flavonoid level detection in mangosteen peel infusion. The materials used for histopathological preparations include 0.9% NaCl, Bouin solution, xylol, toluol, paraffin, eosin Y 1%, Ehrlich hematoxylin, and graded alcohol. For the detection of reactive oxygen species (ROS), nitro blue tetrazolium (NBT) and n, n-dimethylformamide solutions were used. The thiobarbituric acid reactive substances assay kit was used to measure malondialdehyde levels. Furthermore, creatinine and ROS detection were performed using a spectrophotometer (Genesys 10 UV Scanning). Histopathological observations of rat liver and kidney were carried out using a microscope - camera (Leica).

### Feed treatment

Thirty-six rats were divided into six groups: Negative control, positive control (1% H_2_O_2_), and the mangosteen peel infusion treatment groups at various concentrations (1% H_2_O_2_ and 0.25% mangosteen peel infusion; 1% H_2_O_2_ and 0.5% mangosteen peel infusion; 1% H_2_O_2_ and 1% mangosteen peel infusion; or 1% H_2_O_2_ and 2% mangosteen peel infusion). The H_2_O_2_ and mangosteen peel infusion were administered orally at 1 mL each, totaling 2 mL. The treatment was conducted for 2 months, where 1% H_2_O_2_ was administrated orally in the 1^st^ month. In the second, 1% H_2_O_2_ was administrated orally along with mangosteen peel infusion. These treatments were performed simultaneously with feeding time in the morning.

### Creatinine level detection

Creatinine level detection was carried out through a colorimetric method using a spectrophotometer (UV-visible 1800 Shimadzu) with a wavelength of 546 nm. The blank solution was prepared based on Integrated Research and Testing Laboratory protocol as follows: 50 μL of distilled water was mixed with 1000 μL of NaOH, incubated for 5 min and mixed with 250 μL of picric acid. The standard solution was made as follows: 50 μL of standard creatinine was added to 1000 μL NaOH, incubated for 5 min and mixed with 250 μL of picric acid, and standard absorbance set as 1. Furthermore, the creatinine level in the rat was detected through blood serum. The blood sample was collected through the orbital sinus and centrifuged at 10.000 rpm for 10 min to obtain the serum. Afterward, 50 μL of blood serum was added with 1000 μL of NaOH, incubated for 5 min and mixed with 250 μL of picric acid. These samples were then measured by the spectrophotometer at a wavelength of 546 nm.

### ROS level detection

**Figure-1:**
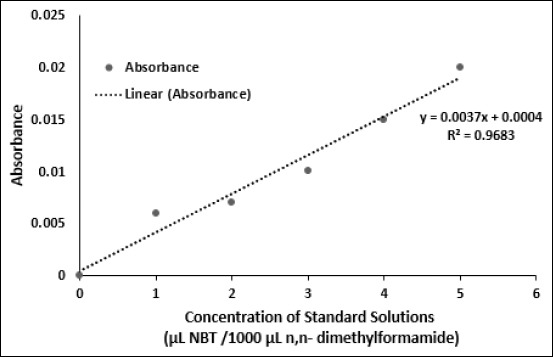
Standard curves used to measure the ROS level in the liver and kidney of rats after H_2_O_2_ induction.

One hundred microliters of mashed rat’s liver or kidney samples were put into a microtiter plate and added with 100 μL of 0.2% NBT solution. They were incubated at room temperature for 30 min. 50 μL of mixture was obtained and added with 1 mL n,n-dimethylformamide. Afterward, the mixture was centrifuged at 10.000 rpm for 2 min. The absorbance was measured using a spectrophotometer (UV-visible 1800 Shimadzu) at a wavelength of 620 nm. The n,n-dimethylformamide was used as a blank solution. Meanwhile, standard solutions were made from various concentrations of NBT mixed with 1000 mL of n,n-dimethylformamide (Figure-1).

### Histological preparation of liver and kidney samples

The rats were euthanized using ketamine 50 mg/mL followed by cervical dislocation. Liver and kidney were obtained and fixed using Bouin solution for ±8 h. The histological preparation was performed using the paraffin method. After fixation, the liver and kidney were dehydrated using graded alcohol and cleared using toluol overnight. The sample was sectioned with a thickness of 6 mm and stained using Hematoxylin-Eosin. Ten samples were selected randomly from each organ for further analysis.

### Kidney histopathological scoring

The scoring of histopathological structures was performed as follows: Two fields of view were taken from each coupe (20 fields of view per 1 mouse). Histopathological scoring was referred to by Khalid *et al*. [15] with an adaptation based on Abdullah *et al*. [5] and Bakour *et al*. [16] (Table-2). The liver morphometry parameters include the diameter of the central vein, diameter of the hepatocyte, length of the hepatocyte, length, and diameter of the sinusoid. These parameters were measured using the Image Raster application.

**Table-2:** Kidney histopathological scoring referred to Khalid *et al*. [15] with an adaptation based on Abdullah *et al*. [5] and Bakour *et al*. [16]

Tissue area	Type of damage	Score
Tubular	No damage	0
	Brush border rupture	1
	Thickening of basal membrane	2
	Hypertrophy of tubular cell	3
Endothelial	No damage	0
	Endothelial swelling	1
	Endothelial rupture	2
	Loss of endothelial	3
Glomerulus	No damage	0
	Thickening of Bowman capsule	1
	Rupture of parietal lamina	2
	Atrophy of glomerulus	3
Interstitial	No damage	0
	Hypertrophy of interstitial tissue	1
	Hemorrhage/congestion	2

### Statistical analysis

Data were analyzed using one-way analysis of variance with SPSS 16.0 (IBM Corp., NY, USA) followed by Duncan’s test at significance p≤0.05.

## Results and Discussion

### Mangosteen peel infusion restored ROS levels of rat’s liver and kidneys after H_2_O_2_ induction

**Figure-2:**
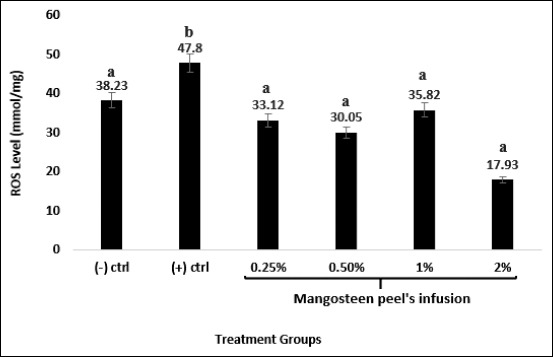
Mangosteen peel’s infusion recovered reactive oxygen species (ROS) levels in the liver during and after H_2_O_2_ treatment. Negative control group: Distilled water, positive control group: 1% H_2_O_2_ and mangosteen peel infusion treatment groups at various concentration alongside 1% H_2_O_2_ treatment. ROS levels were detected during the last day of treatment using a spectrophotometry method. n=6. Different letters on the graph showed significant difference compared to negative control group at p≤0.05.

The results showed that H_2_O_2_ treatment for 2 months elevated the ROS levels of rat’s liver. Whereas, treatment with mangosteen peel’s infusion reduced the ROS levels caused by H_2_O_2_. Mangosteen peel’s infusion up to a concentration of 1% could restore the ROS to a basal level (ROS level of the negative control group was used as basal level). At a concentration of 2%, mangosteen peel infusion reduced the ROS level more significantly than the basal level (Figure-2).

**Figure-3:**
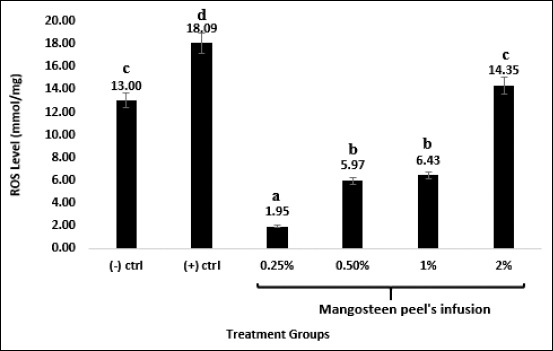
Mangosteen peel’s infusion reduced reactive oxygen species (ROS) levels in the kidney of Wistar rats during and after H_2_O_2_ treatment. Negative control group: Distilled water, positive control group: 1% H_2_O_2_, and mangosteen peel infusion treatment groups at various concentration along with 1% H_2_O_2_ treatment. ROS levels were detected during the last day of treatment using a spectrophotometry method. n=6. Different letters on the graph showed significant difference compared to negative control group at p≤0.05.

H_2_O_2_ treatment also significantly induced the elevation of ROS levels in the kidneys of rats in the positive control group. Meanwhile, mangosteen peel infusion also normalizes ROS levels caused by H_2_O_2_ induction (Figure-3).

Naturally, ROS plays a defensive role against pathogens in the body. However, high amounts of ROS result in an imbalance situation that causes peroxidation and cellular degeneration [17]. There are several types of radical ROS, one of which is hydroxyl radicals (OH•). OH• is produced by the interactions between H_2_O_2_ and ferrous ions within the body [18].

Wijeratne *et al*. [19] stated that H_2_O_2_ treatment increases oxidative stress by decreasing the superoxide dismutase enzyme (H_2_O_2_-breaking down enzyme) and increasing hydroxyl levels. The results of this study showed that the mangosteen peel infusion, especially at a concentration of 2% normalized ROS levels in the liver and kidney of rats during and after H_2_O_2_ induction. This process may also be supported by certain compounds within the mangosteen peel infusion, such as flavonoid, saponin, alkaloid, triterpenoid, tannin, and polyphenol.

The flavonoid of mangosteen peel possesses the B-ring-dihydroxyl which has a great capacity to inhibit ROS produced by binding with Fe ions that catalyze the production of •OH. This flavonoid also enhances the rigidity of the membrane cells and interacts with the polar head of the membrane to protect it from oxidative damage [20].

Lobo *et al*. [21] stated that antioxidants inhibit ROS formation through three steps. First, antioxidants convert hydroperoxides and hydrogen peroxide to alcohol and water, thereby decreasing free-radical formation. Second, antioxidants may arrest the initial chain reaction and/or break further chain reaction. Third, antioxidants break down and dispose of the radical products. The elevation of ROS levels after antioxidant treatment may occur because antioxidants can be pro-oxidants, which were detected as ROS [22]. Therefore, when the ROS level of the kidneys was tested, the positive control group showed the highest levels because the resonance reaction (chain reaction occurred repeatedly) caused huge amounts of ROS. The results showed that mangosteen peel infusion at a concentration of 0.25% was enough to neutralize ROS in the kidneys (Figure-3). Poljsak *et al*. [23] stated that the excessive intake of antioxidants decreases the levels of free radicals but interferes with the immune system of the body. Excessive intake of antioxidants also causes the production of pro-oxidants which are detected as ROS.

Due to the observation that mangosteen peel infusion restores ROS levels in the liver and kidney of the rats, kidney function was further evaluated by determining the creatinine level. Giknis and Clifford [24] stated that the physiological creatinine range is 0.20-0.60±0.10 mg/dL. The results showed that rat creatinine levels changed during the test duration. Creatinine levels decreased after H_2_O_2_ induction before treatment using mangosteen peel infusion. However, the results showed that mangosteen peel infusion treatments tend to recover the creatinine levels similar to the negative control groups. The creatinine level of the positive control group was lower compared to the other groups, although there was no significant difference compared to the negative control. Moreover, a 2% treatment of mangosteen peel infusion showed significant recovery from the effect of H_2_O_2_ induction (Table-3).

**Table-3:** Rat’s creatinine levels before H_2_O_2_ induction (I), after H_2_O_2_ induction (II), and after mangosteen peel infusion treatment (III) (mg/mL).

Groups	Creatinine level (mg/mL)		

	I^x^	II	III^x^
Negative control	0.35± 0.074^a^	0.33± 0.027^a^	0.36± 0.059^ab^
Positive control (1% H_2_O_2_)	0.34± 0.063^a^	0.29± 0.075^bc^	0.29± 0.22a
1% H_2_O_2_ and 0.25% mangosteen peel infusion	0.36± 0.073^a^	0.25± 0.044^ab^	0.35± 0.031^ab^
1% H_2_O_2_ and 0.5% mangosteen peel infusion	0.36± 0.074^a^	0.26± 0.034^ab^	0.35± 0.039^ab^
1% H_2_O_2_ and 1% mangosteen peel infusion	0.35± 0.071^a^	0.31± 0.053^bc^	0.36± 0.042^b^
1% H_2_O_2_ and 2% mangosteen peel infusion	0.38± 0.037^a^	0.22± 0.056^b^	0.35± 0.071^a^

Notes: x) I and III had significant difference with II (p≤0.05). The different superscript letters in the same column showed a significant difference (p≤0.05)

Creatinine is the final product of creatinine metabolism, and creatinine synthesis occurs within the liver. Creatinine levels are one of the parameters to detect kidney function. Risk levels in humans occur when there is an increase in serum creatinine about 1.5 times from the baseline. The condition becomes critical where there is a two-fold increase in serum creatinine compared to the baseline. Furthermore, kidney failure occurs when there is an increase in serum creatinine three-fold and above the baseline [25]. In this research, it was observed that the creatinine serum level depletion was caused by ROS which influenced its metabolism.

High serum creatinine levels indicate failure of creatinine filtration in the kidney. However, both the decrease and increase of creatinine levels are not always caused by poor kidney function but also due to certain factors such as muscle mass change or drugs [26].

### Mangosteen peel infusion improved the histopathological structure of the liver and kidney of Wistar rats after H_2_O_2_ induction

In this study, the histological morphometry of the liver was ascertained to assess its condition. The diameter of the central vein, sinusoid, and hepatocytes was measured. Bhadoria *et al*. [27] stated that radiation caused the reduction of hepatocyte size and increased sinusoid and central vein size. This study agreed with Abozid *et al*. [28] which observed that H_2_O_2_ caused liver cell damage which changed hepatocyte sizes and caused necrosis. The results of this study showed that 1% of H_2_O_2_ treatment significantly increased the diameter of the central vein and sinusoid (Table-4). Kleiner [29] observed liver injury such as congestion or damage of central vein and sinusoidal dilatation. Furthermore, Hoehme *et al*. [30] stated that the blood exchanges substances with hepatocytes during its flow from the sinusoid into the central vein. These substances contain all of the metabolic results including toxic substances. In the cirrhotic liver, sinusoidal dilatation is found in stress areas [31]. Therefore, sinusoidal dilatation is an appropriate parameter to evaluate the liver injury, as the dilatation was caused by hepatic necrosis. Necrotic hepatocytes have irregular forms and messy structures within the lobes. Therefore, the sinusoid located close to the damaged hepatocyte was affected. The dilatation of the sinusoid is also caused by the high amount of toxicants in the blood passing through the sinusoid into the central vein [29]. The treatment of mangosteen peel infusion up to 2% may improve the central vein diameter similar to that of the control, while the treatment of mangosteen peel infusion up to 1% may improve sinusoid length diameter. The treatment of mangosteen peel infusion at 2% concentration instead, caused an increase of sinusoid diameter. However, both H_2_O_2_ and mangosteen peel infusion did not influence the hepatocyte diameter (Table-4).

**Table-4:** Liver morphometry in each treatment group after H_2_O_2_ and mangosteen peel infusion for 2 months.

Treatment	Parameter (µm)±standard error					

	Diameter of the length central vein	Diameter of the width central vein	Diameter of the length sinusoid	Diameter of the width sinusoid	Diameter of the length hepatocytes	Diameter of the width hepatocytes
Negative control	0.055±0.016^a^	0.025±0.003^a^	0.033±0.001^b^	0.009±0.0004^a^	0.013±0.001^b^	0.015±0.005^b^
Positive control (1% H_2_O_2_)	0.136±0.040^b^	0.044±0.006^ab^	0.042±0.005^ab^	0.012±0.001^a^	0.012±0.002^b^	0.009±0.001^ab^
1% H_2_O_2_ and 0.25% mangosteen peel infusion	0.083±0.013^ab^	0.043±0.004^ab^	0.036±0.003^a^	0.011±0.001^a^	0.008±0.002^a^	0.006±0.004^a^
1% H_2_O_2_ and 0.5% mangosteen peel infusion	0.054±0.003^a^	0.033±0.003^a^	0.032±0.003^ab^	0.011±0.0006^a^	0.014±0.004^b^	0.007±0.0002^a^
1% H_2_O_2_ and 1% mangosteen peel infusion	0.059± 0.007^a^	0.043±0.005^ab^	0.040±0.003^ab^	0.011±0.0008^a^	0.012±0.0005^b^	0.008±0.0006^ab^
1% H_2_O_2_ and 2% mangosteen peel infusion	0.065±0.014^a^	0.065±0.014^b^	0.054±0.01^a^	0.054±0.01^b^	0.012±0.0005^b^	0.008±0.0006^ab^

**Figure-4:**
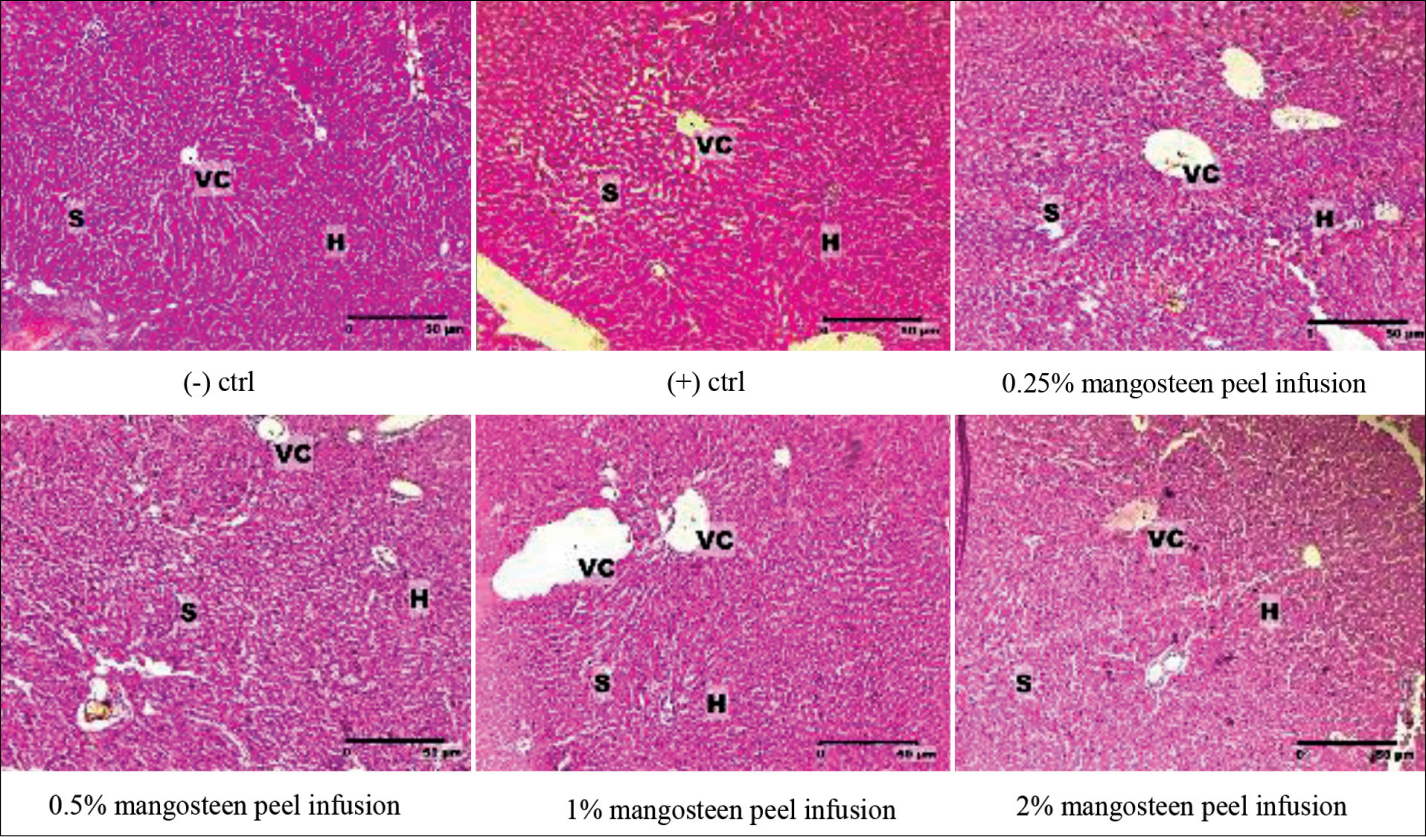
Mangosteen peel infusion improved the histopathological structure of the liver of Wistar rats induced by H_2_O_2_. The rats were treated with 1% of H_2_O_2_ for 1 month followed by mangosteen peel infusion at various concentrations+1% H_2_O_2_ for the next 1 month. (-) ctrl, (+) ctrl (1 mL 1% H_2_O_2_); 0.25% mangosteen peel infusion +1 mL 1% H_2_O_2_; 0.5% mangosteen peel infusion+1 mL 1% H_2_O_2_; 1% mangosteen peel infusion+1 mL 1% H_2_O_2_; and 2% mangosteen peel infusion+1 mL 1% H_2_O_2_. VC=Central vein; H=Hepatocytes; S=Sinusoids. Stained using Hematoxylin-Eosin. Scale bar: 50 mm.

Alongside the evaluation of the liver’s morphometry, the effect of H_2_O_2_ induction, as well as mangosteen peel infusion on the histopathological structure of rat liver, was also determined. The results showed that H_2_O_2_ induced vein dilatation and increased the size of sinusoids and hepatocytes which signified cellular and organ damage. In Figure-4, the changes in the liver structure of the test animals can be observed histologically, as the negative control group showed normal structure and size. The positive control group showed an increased distance between the sinusoids. The central and sinusoid veins were enlarged and reduced in size. Furthermore, treatment with mangosteen peel infusion at a concentration of 0.25% did not show a significant difference in histological structure in the positive control group. The reduction in central vein size was observed in the treatment with mangosteen peel infusion at 2% concentration.

The histopathological effect of H_2_O_2_ induction followed by the mangosteen peel infusion on the kidneys of Wistar rats was examined in the tubular, endothelial, glomerular, and interstitial areas. The scoring of histopathological structure is in line with Khalid *et al*. [15] with modification based on Abdullah *et al*. [5] and Bakour *et al*. [16] (Table-2).

**Figure-5:**
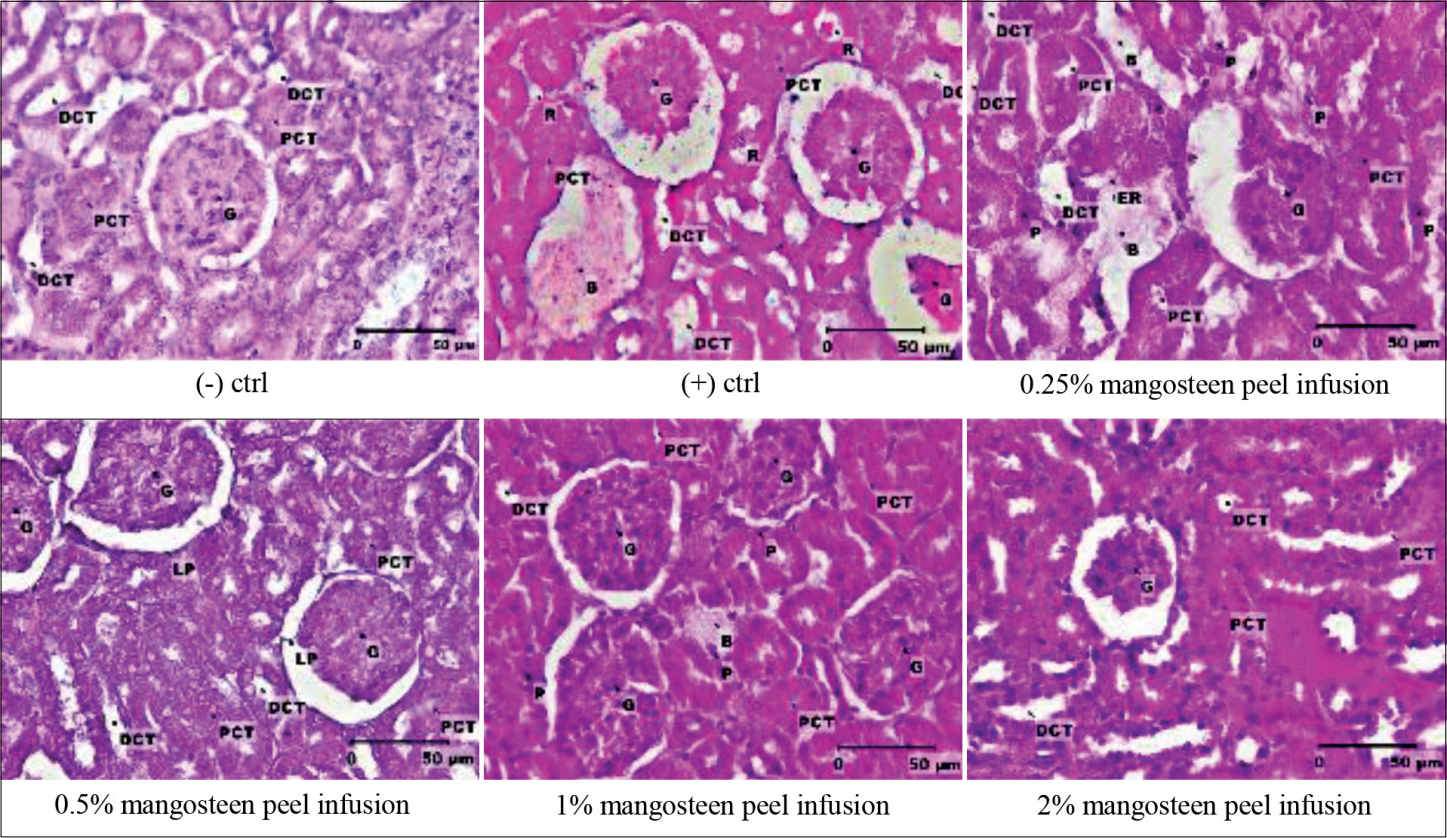
Mangosteen peel infusion improved the histopathological structure of the kidney of Wistar rats induced by H_2_O_2_. The rats were treated with 1% of H_2_O_2_ for 1 month followed by mangosteen peel infusion at various concentrations+1% H_2_O_2_ for the next 1 month. (-) ctrl, (+) ctrl (1 mL H_2_O_2_ 1%); 0.25% mangosteen peel infusion +1 mL H_2_O_2_ 1%; 0.5% mangosteen peel infusion+1 mL H_2_O_2_ 1%; 1% mangosteen peel infusion+1 mL H_2_O_2_ 1%; and 2% mangosteen peel infusion+1 mL H_2_O_2_ 1%. G=Glomerulus, PCT=Proximal contral tubule, DCT=Distal contral tubule, LP=The lamina parietalis, R=Rupture, P=Picnosis, B=Bleeding, Er=Rupture of the endothelium. Stained using Hematoxylin-Eosin. Scale bar: 50 μm.

The results showed that 1% H_2_O_2_ caused damage to the kidneys. Furthermore, H_2_O_2_ induction caused various damages in the renal cortex areas especially in tubular, glomerular, and interstitial areas, but did not affect endothelial cells. H_2_O_2_ compound caused brush border rupture, thickening of basal membrane, cellular hypertrophy in the tubular area, and atrophy of glomerulus. In the interstitial area, H_2_O_2_ treatment caused hypertrophy of interstitial tissue as well as hemorrhage or congestion. However, mangosteen peel infusion at the lowest concentration of 0.25% did not have any visible or significant effect to ameliorate H_2_O_2_ induction. This was also observed in H_2_O_2_ groups, especially at the tubular and glomerular areas. Instead of damages in the interstitial area as observed in the H_2_O_2_ group, the endothelial rupture was observed in 0.25% of the mangosteen peel infusion group. Moreover, brush border rupture, cellular hypertrophy in tubular area, thickening of Bowman capsule, and rupture of the parietal lamina in the glomerulus was observed in the group treated with 0.5% of mangosteen peel infusion. The histopathological structure changed alongside an increase in mangosteen peel infusion concentration. At a 1% concentration of mangosteen peel infusion, brush border rupture was observed in the tubular area, atrophy of glomerulus, and hemorrhage or congestion in the interstitial area. Meanwhile, at a concentration of 2% mangosteen peel infusion, only tubular border rupture and atrophy of the glomerulus were observed. These results signified that treatment of mangosteen peel infusion after and along with H_2_O_2_ induction may improve the histological structure of the kidney. The higher the concentration of mangosteen peels infusion, the more improved histological structure in the kidneys of the rats (Table-5 and Figure-5). These results were consistent with Widowati *et al*. [11] which stated that treatment of mangosteen peel extract could prevent ongoing damage to the kidney because mangosteen peel contains flavonoids that reduce hydroxyl and reduce the capacity of ferrous ions which cause the production of ROS.

**Table-5:** Histopathological scoring results of rats’ kidney.

Type of tissues	Type of damages	Score	Control	1% H_2_O_2_	1% H_2_O_2_ and 0.25% mangosteen peel infusion	1% H_2_O_2_ and 0.5% mangosteen peel infusion	1% H_2_O_2_and 1% mangosteen peel infusion	1% H_2_O_2_and 2% mangosteen peel infusion
Tubular	No damage	0	-	-	-	-	-	-
	Brush border rupture	1	1	1	1	1	1	1
	Thickening of basal membrane	2	-	2	-	-	-	-
	Hypertrophy of tubular cell	3	-	3	3	3	-	-
Endothelial	No damage	0	0	0	-	0	0	0
	Endothelial swelling	1	-	-	-	-	-	-
	Endothelial rupture	2	-	-	2	-	-	-
	Loss of endothelial cells	3	-	-	3	-	-	-
Glomerulus	No damage	0	0	-	-	-	-	-
	Thickening of Bowman capsule	1	-	-	-	1	-	-
	Rupture of parietal lamina	2	-	-	-	2	-	-
	Atrophy of glomerulus	3	-	3	3	-	3	3
Interstitial	No damage	0	0	-	0	0	-	0
	Hypertrophy of interstitial tissue	1	-	1	-	-	-	-
	Hemorrhage/congestion	2	-	2	-	-	2	-

ROS has the potential to damage nearly all cellular macromolecules in the body. In normal amounts, ROS binds with endogen antioxidants, forming non-toxic substances. Endogen antioxidants are produced in constant amounts and do not increase. However, when the amount of ROS exceeds the capacity, endogen antioxidant levels decrease, and continue to run out. When there are no antioxidants, ROS accumulates and disturbs metabolism through caspase activation, and the lysosome enzymes initiate apoptosis especially on proximal tube cells. Apoptosis causes changes in the superficial filtration structure and filtration coefficient, leading to kidney damage [32-35]. Moreover, Kumari *et al*. [36] observed that ROS caused hypoxia which led to cellular damage because of unstable homeostasis. The results of this study also agree with Ratliff *et al*. [37] which stated that kidney cell damage caused by lipid peroxidation induced ROS. A continuous lipid peroxidation damages the cells rapidly as it ruptures the cell barrier. This leads to apoptosis, as observed in the positive control group (Figure-5).

This study was consistent with previous research which stated that H_2_O_2_ treatment caused kidney bleeding in mice [16]. This bleeding was due to the dilation or fracture of the blood vessels. Abdullah *et al*. [5] concluded that H_2_O_2_ treatment caused glomerular atrophy and hypertrophy in the parietal lamina. These occurred because cells have to adapt to environmental changes, both chemically and physically [38].

From the results, it was observed that mangosteen peel infusion helps to repair damaged cells. These results are also supported by Putri *et al*. [39] who observed that mangosteen peel increased cell protection and repair due to its flavonoid content such as xanthones, tannins, and catechins. Jittiporn *et al*. [40] stated that α-mangostin and various polyphenols in the aqueous extract of mangosteen prevent free-radical formation. This protects human endothelial cell membranes from the adverse effect of H_2_O_2_.

## Conclusion

The collective results showed that mangosteen peel infusion restores the ROS levels of both the liver and kidneys of female Wistar rats. Furthermore, the infusion of mangosteen peel minimizes the liver and kidney damage of female Wistar rats caused by H_2_O_2_ induction. About 2% of mangosteen peel infusion was also observed to be suitable for use as a therapeutic agent to reduce the adverse effects of H_2_O_2_.

## Authors’ Contributions

HTS, JRAR, SAS, and AN: Planned the study. HTS, SAS, and AN: Drafted and edited the manuscript. All authors read and approved the final manuscript.
